# CO_2_ Signaling through the Ptc2-Ssn3 Axis Governs Sustained Hyphal Development of Candida albicans by Reducing Ume6 Phosphorylation and Degradation

**DOI:** 10.1128/mBio.02320-18

**Published:** 2019-01-15

**Authors:** Yang Lu, Chang Su, Shatarupa Ray, Yuncong Yuan, Haoping Liu

**Affiliations:** a Hubei Key Laboratory of Cell Homeostasis, College of Life Sciences, Wuhan University, Wuhan, China; b Department of Biological Chemistry, School of Medicine, University of California, Irvine, California, USA; University of Texas Health Science Center at San Antonio; University of Texas Health Science Center

**Keywords:** *Candida albicans*, Ssn3/Cdk8, Ume6, carbon dioxide signaling, hyphal development

## Abstract

Candida albicans is the most common cause of invasive fungal infections in humans. Its ability to sense and adapt to changing carbon dioxide levels is crucial for its pathogenesis. Carbon dioxide promotes hyphal development. The hypha-specific transcription factor Ume6 is rapidly degraded in air, but is stable under physiological CO_2_ and hypoxia to sustain hyphal elongation. Here, we show that Ume6 stability is regulated by two parallel E3 ubiquitin ligases, SCF^Grr1^ and Ubr1, in response to CO_2_ and O_2_, respectively. To uncover the CO_2_ signaling pathway that regulates Ume6 stability, we performed genetic screens for mutants unable to respond to CO_2_ for sustained filamentation. We find that the type 2C protein phosphatase Ptc2 is specifically required for CO_2_-induced stabilization of Ume6 and hyphal elongation. In contrast, the cyclin-dependent kinase Ssn3 is found to be required for Ume6 phosphorylation and degradation in atmospheric CO_2_. Furthermore, we find that Ssn3 is dephosphorylated in 5% CO_2_ in a Ptc2-dependent manner, whereas deletion of *PTC2* has no effect on Ssn3 phosphorylation in air. Our study uncovers the Ptc2-Ssn3 axis as a new CO_2_ signaling pathway that controls hyphal elongation by regulating Ume6 stability in C. albicans.

## Abstract (precis)

The capacity to sense and adapt to changing carbon dioxide levels is crucial for all organisms. In fungi, CO_2_ is a key determinant involved in fundamental biological processes, including growth, morphology, and virulence. In the pathogenic fungus Candida albicans, high CO_2_ is directly sensed by adenylyl cyclase to promote hyphal growth. However, little is known about the mechanism by which hyphal development is maintained in response to physiological levels of CO_2_. Here we report that a signal transduction system mediated by a phosphatase-kinase pair controls CO_2_-responsive Ume6 phosphorylation and stability that in turn dictate hyphal elongation. Our results unravel a new regulatory mechanism of CO_2_ signaling in fungi.

## INTRODUCTION

Candida albicans is a common opportunistic fungal pathogen of humans. As a part of the commensal microbiota, C. albicans is a benign inhabitant of the gastrointestinal and genitourinary tracts most of the time. However, it can infect sites ranging from the skin and the oral and vaginal mucosa to deep tissues if host or environmental factors are permissive (1). Disseminated invasive candidiasis has an estimated mortality rate of 40%, even with the use of antifungal drugs (2). With the limited types of antifungal drugs available and rising populations of susceptible patients, there is a pressing need for understanding mechanisms of *Candida* pathogenesis in order to develop new approaches for treating invasive candidiasis.

Numerous traits that contribute to virulence have been documented for C. albicans, and among the most prominent is its ability to grow either as a unicellular budding yeast or in filamentous forms (3). Unlike dimorphic fungal pathogens of humans (e.g., Histoplasma capsulatum, Paracoccidioides brasiliensis, and *Talaromyces* [formerly *Penicillium*] *marneffei*) that normally grow in filamentous forms outside the human body but convert to yeast form in human tissues (4), C. albicans is able to switch reversibly between yeast, pseudohyphae, and hyphal growth forms, and is found in both yeast and filamentous forms in the host (5). The hyphal form plays key roles in the infection process, and has a variety of specific properties linked to virulence, including adherence (6, 7), secretion of hydrolases (8), and candidalysin (9), to damage host cells. Hypha-specific genes *UME6* and *HGC1* are regulators of hyphal transcription and morphogenesis (10–12). Levels of the transcription factor Ume6 control the levels and duration of hypha-specific transcription (13). The yeast-to-hypha transition requires initiation and then maintenance. Hyphal initiation requires a rise in temperature to 37°C and release from quorum sensing molecules, such as farnesol, to temporarily clear the major repressor of hyphal morphogenesis, Nrg1 (14, 15). Hyphal maintenance requires active sensing of the surrounding environment. Nutrient limitation, serum, or *N*-acetylglucosamine activates the expression of transcription factor Brg1 to recruit the Hda1 histone deacetylase to promoters of hypha-specific genes, leading to nucleosome repositioning, obstruction of Nrg1 binding sites, and sustained hyphal development (16). In parallel to the nutrient-responsive chromatin-remodeling pathway, the combination of hypoxia and high CO_2_, but neither condition alone, maintains hyphal elongation by stabilizing the transcription activator Ume6, leading to sustained hyphal development through a positive feedback loop in which Ume6 activates its own transcription (17). The Ume6 stabilization and chromatin-remodeling pathways act in parallel to control hyphal development and virulence during disseminated infection. Ofd1, a prolyl 4-hydroxylase-like 2-oxoglutarate-Fe(II) dioxygenase, acts as an oxygen sensor that regulates Ume6 stability in response to hypoxia (17, 18). Ume6 stability in C. albicans is also controlled by the level of CO_2_. However, the signaling pathway for the CO_2_-responsive Ume6 stabilization in hyphal elongation remains elusive in C. albicans.

Reversible protein phosphorylation is a key protein modification involved in the regulation of numerous physiological processes. It is an extremely common event in signal transduction, and it is considered the main mechanism of posttranslational modification leading, for instance, to a change in enzyme activity. The phosphorylation state of a protein results from the balance of protein kinases and protein phosphatase activities. Ssn3 and its cyclin Ssn8, along with the cofactors Srb8/Med12 and Srb9/Med13, form the kinase submodule of the RNA polymerase-associated mediator complex that functions as a bridge between basal transcription machinery and gene-specific transcriptional factors (19). Ssn3 has also emerged as a key regulator of multiple transcriptional programs linked to nutrient sensing and differentiation control in Saccharomyces cerevisiae (20). Type 2C protein phosphatases (PP2Cs) remove phosphate from Ser and Thr residues (21), and are widely represented in bacteria, fungi, plants, insects, and mammals (22, 23). In S. cerevisiae, there are seven PP2C-encoding genes (24–26), which share a conserved PP2C domain. Among them, Ptc2 serves to limit the maximum of activation of Hog1 (27). It also regulates negatively the unfolded-protein response through dephosphorylation of the Ser/Thr protein kinase Ire1 (28). In addition, Ptc2 and Ptc3 have been implicated in the regulation of progression through the cell cycle since they are capable of dephosphorylating Cdc28 at Thr169 (24), a residue essential for its activity as a cyclin-dependent kinase. Ptc2 in C. albicans shares a functional role with S. cerevisiae Ptc2, as a *Captc2* mutant displays hypersensitivity to the genotoxic stress-inducing agents methyl methanesulfonate and hydroxyurea (29). It has been reported that Ppg1, a putative type 2A-related protein phosphatase (PP2A), is important for C. albicans filament extension, invasion, and virulence (30). Relatively little is known regarding PP2C in the regulation of hyphal development in response to the changing environments in C. albicans.

CO_2_ serves basic metabolic functions as both a building block and a waste product and thus plays a central role in the carbon cycle (31, 32). It is not a major component of the atmosphere, comprising only 0.0365%, but its levels are substantially higher in our bloodstream and tissues, where as an end product of respiration it is found at levels of roughly 5%. Elevated CO_2_ levels induce virulence factors such as capsule biosynthesis and filamentation in opportunistic fungal pathogens Cryptococcus neoformans (33, 34) and Candida albicans (35) through adenylyl cyclase-dependent signaling pathways (36, 37). CO_2_/HCO_3_^−^ also signals independently of adenylyl cyclase to regulate levels of carbonic anhydrase (38, 39) and promote cell-fate transition (40). Here, we report that a high concentration of CO_2_ triggers the dephosphorylation of Ssn3 by Ptc2 that in turn reduces Ume6 phosphorylation and prevents its degradation, leading to sustained hyphal development. Our results demonstrate that the Ptc2-Ssn3 axis represents a new regulatory module of CO_2_ signaling.

## RESULTS

### Two distinct E3 ubiquitin ligases control Ume6 stability in response to hypoxia and high CO_2_, respectively.

**FIG 1:**
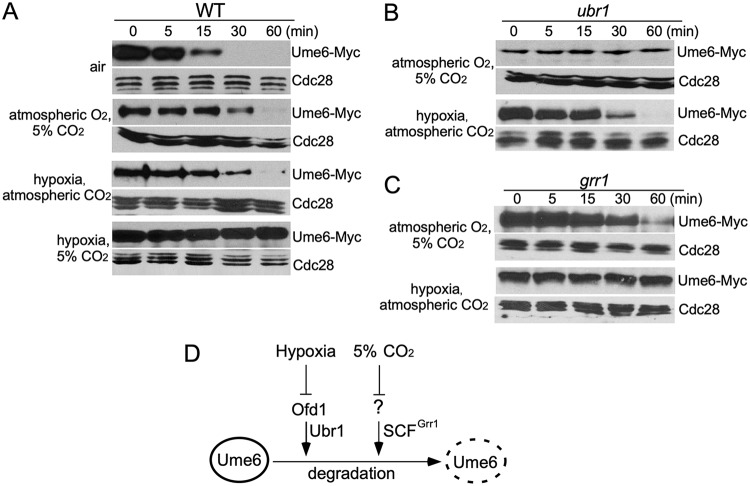
Two distinct E3 ubiquitin ligases control Ume6 degradation in response to O_2_ and CO_2_. (A) Ume6 stability was monitored by *MET3* promoter shutdown assay. Wild-type C. albicans cells containing Ume6*_C778/785S_*-Myc (shown as Ume6-Myc) under the regulation of the *MET3* promoter were grown in SCD (−Met, −Cys) for 2 h to induce their expression at room temperature. Twenty-five milliliters of medium was transferred from the culture to a petri dish (150 × 15 mm) and incubated in air, hypoxia (0.2% O_2_), 5% CO_2_, or hypoxia plus 5% CO_2_ as indicated at 30°C for 4 h. Methionine at 5 mM was then added to shut off the promoter. Aliquots were harvested at times indicated for the anti-Myc Western blot analysis. (B) Ubr1 is critical for O_2_-responsive Ume6 degradation. Ume6 stability was monitored by *MET3* promoter shutdown assay in the *ubr1* mutant under indicated conditions. (C) Ume6 degradation under atmospheric CO_2_ requires the SCF^Grr1^ E3 ligase. Ume6 stability was monitored by *MET3* promoter shutdown assay in the *grr1* mutant under indicated conditions. (D) Model for regulation of Ume6 degradation by oxygen and CO_2_. Ofd1 mediates the regulation of Ume6 degradation by E3 Ubr1 in response to oxygen, and CO_2_ regulates Ume6 degradation by SCF^Grr1^ through an unknown mechanism.

We have demonstrated that Ume6 was continuously degraded in air, but stable in hypoxia combined with 5% CO_2_ to promote hyphal elongation in C. albicans (17). Both low oxygen and high CO_2_ contributed to Ume6 stabilization, but neither alone was sufficient (Fig. 1A). Ofd1 regulates Ume6 stability in response to oxygen concentration (17). In Schizosaccharomyces pombe, Ofd1-mediated protein degradation in O_2_ requires the E3 ubiquitin ligase Ubr1 (41). To investigate whether a similar regulation exists in C. albicans, we examined Ume6 turnover in hypoxia (0.2% O_2_) or 5% CO_2_ by promoter shutoff assays. A gene encoding Ume6*_C778/785S_*, which had the Cys778 → Ser and Cys785 → Ser substitutions in the Gal4 DNA binding domain of Ume6, was expressed under the control of the *MET3* promoter. The DNA binding domain of Ume6 was mutated in the construct to disrupt its affinity for DNA as *MET3* expression could not be shut off completely when wild-type Ume6 was expressed (17). If Ubr1 is responsible for Ume6 degradation in O_2_, Ume6 is expected to be stable in atmospheric O_2_ plus 5% CO_2_. Indeed, we found that Ume6 was stable in the *ubr1* mutant under this condition (Fig. 1B). Therefore, Ume6 degradation in atmospheric O_2_ depends on the Ubr1 ubiquitin ligase. Importantly, deletion of *UBR1* could not block Ume6 degradation in atmospheric CO_2_ (Fig. 1B), suggesting that Ume6 degradation in response to CO_2_ concentration is controlled by additional E3 ligases. We have previously shown that stabilization of the hypha-specific G_1_-like cyclin Hgc1, like Ume6, requires both hypoxia and 5% CO_2_ (17). Hgc1 is unstable when expressed in S. cerevisiae, and Hgc1 degradation was blocked by deleting *GRR1* (42), which encodes the F-box protein of the SCF^Grr1^ ubiquitin ligase complex. However, Hgc1 degradation was not blocked in C. albicans *grr1* mutants in air (42, 43). We found that the C. albicans *grr1* mutant hampered Hgc1 degradation only in hypoxia, and Hgc1 stability in the *grr1* mutant was not affected by CO_2_ levels anymore (see Fig. S1 in the supplemental material). Thus, CO_2_-responsive Hgc1 degradation depends on the SCF^Grr1^. Given that both hyphal regulators, Ume6 and Hgc1, are stable in hypoxia plus 5% CO_2_, one would predict that the SCF^Grr1^ is also responsible for Ume6 degradation in response to CO_2_ level. As shown in Fig. 1C, the *grr1* mutant blocked Ume6 degradation in hypoxia regardless of CO_2_ levels. The O_2_-sensitive and CO_2_-insensitive Ume6 stability in the *grr1* mutant further supports the specificity of the SCF^Grr1^ for CO_2_-responsive Ume6 degradation. Together, our data suggest that Ume6 stability in C. albicans is regulated by two parallel E3 ubiquitin ligases under the control of specific signaling pathways in response to O_2_ and CO_2_, respectively (Fig. 1D).

10.1128/mBio.02320-18.1 FIG S1 Hgc1 is stabilized in hypoxia in the *grr1* mutant. Stability of Hgc1 was monitored by a *MAL2* promoter shut down in air or in hypoxia (0.2% O_2_). Western blot analysis in C. albicans wild-type cells expressing Myc-Hgc1 from the *MAL2* promoter. Download FIG S1, PDF file, 0.1 MB. Copyright © 2019 Lu et al. 2019 Lu et al. This content is distributed under the terms of the Creative Commons Attribution 4.0 International license.

### Ptc2 is required for CO_2_-induced Ume6 stabilization.

**FIG 2:**
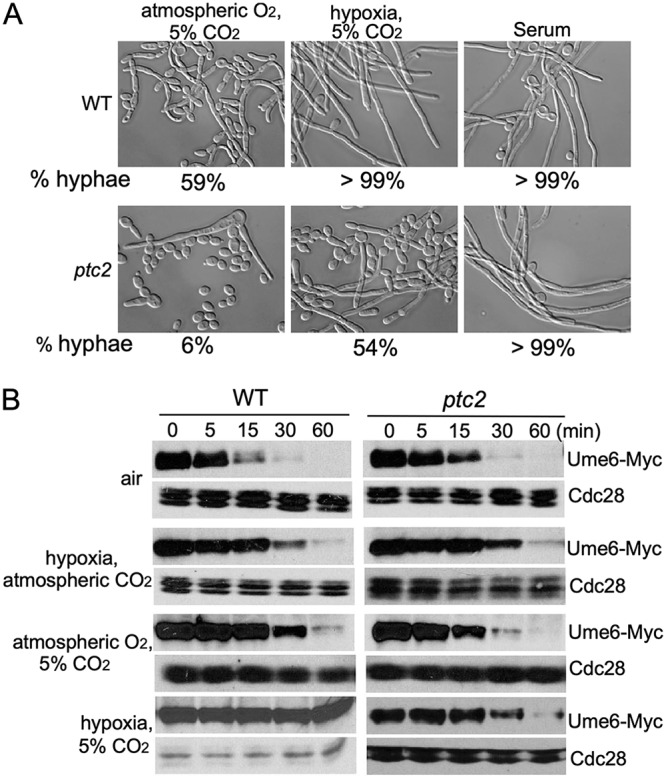
Ptc2 is critical for CO_2_-induced hyphal elongation. (A) Overnight cultures of wild-type and *ptc2* mutant were diluted into YEPSucrose medium at 37°C. One-third of the samples were put into the CO_2_ incubator immediately, and cell morphology was examined after incubation for 5 h (left panels). One-third of the samples were put into the hypoxic chamber immediately and incubated for 12 h to analyze cell morphology in hypoxia plus 5% CO_2_ (middle panels). Ten percent serum was added to the other samples. Photographs were taken after 3.5 h of incubation (right panels). The percentage of cells forming hyphae was determined by counting at least 200 cells/sample. The data show the average from three independent experiments. The cells which had a length/width ratio of >4.5 and characteristic shape were considered hyphae. (B) Ume6 protein cannot be stabilized in 5% CO_2_ in *ptc2* mutant. The protein stability of Ume6 in wild type and *ptc2* mutant was monitored by *MET3* promoter shutdown.

To identify the CO_2_ signaling pathway that regulates Ume6 stability, genetic screens were carried out to identify genes important for Ume6 stability in high CO_2_. We performed a screen with a knockout library of 674 unique genes in C. albicans (44) for mutants that are unable to sustain hyphal elongation in YEP+sucrose under hypoxia plus 5% CO_2_ (see Materials and Methods). The *ptc2* mutant (type 2C protein phosphatase) was found to have impaired hyphal elongation under 5% CO_2_, but normal hyphal elongation in serum (Fig. 2A). Most of the *ptc2* mutant cells converted to yeast under 5% CO_2_. However, about 50% cells of *ptc2* mutant could form hyphae in hypoxia plus 5% CO_2_ (Fig. 2A), suggesting that deletion of *PTC2* had no detectable defect in hypoxia-induced hyphal elongation. We also screened the GRACE library, a nonredundant library containing a total of 2,357 different mutants (45). *ptc2* was the only mutant found specifically defective in CO_2_-induced hyphal elongation. These results indicated that Ptc2 is specifically required for hyphal elongation in high CO_2_. Correspondingly, Ume6 in the *ptc2* mutant was unstable under hypoxia plus 5% CO_2_, less stable than in the WT strain under 5% CO_2_, and the same as in WT under hypoxia (Fig. 2B). The stability of Ume6 in the *ptc2* mutant in 5% CO_2_ was similar to that in wild-type cells in air (Fig. 2B). Therefore, the defect of *ptc2* mutant in Ume6 stability and hyphal elongation is detectable only in the presence of 5% CO_2_.

### Ssn3 promotes Ume6 degradation in atmospheric CO_2_.

**FIG 3:**
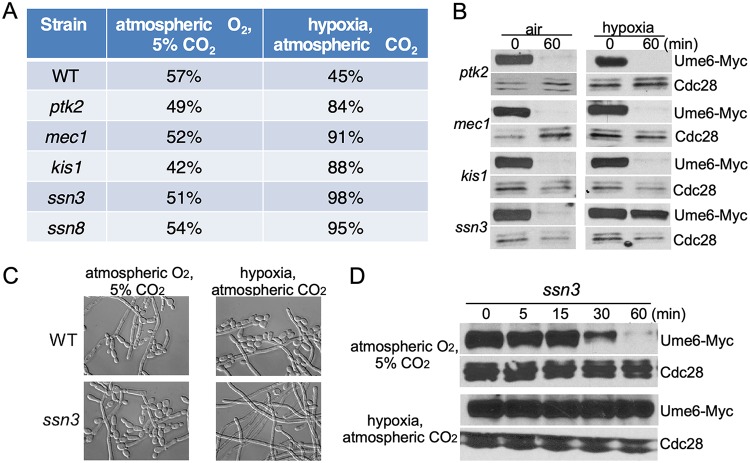
Ssn3 is required for Ume6 degradation in atmospheric CO_2_. (A) The percentage of cells forming hyphae of indicated strains was determined as described for Fig. 2A. (B) The protein stability of Ume6 in indicated strains was monitored by *MET3* promoter shutdown. (C) Morphology analysis of wild-type and *ssn3* mutant cells was performed as described in Fig. 2A. (D) The protein stability of Ume6 in *ssn3* mutant was monitored by *MET3* promoter shutdown under 5% CO_2_ or hypoxia condition.

Ptc2 is a type 2C protein phosphatase (PP2C) in C. albicans (29). In S. cerevisiae, Ptc2 dephosphorylates Hog1, Ire1, and Cdc28, which are involved in the regulation of osmostress response, unfolded protein response, and cell cycle progression, respectively (24, 27, 28). So far, all known substrates of Ptc2 are kinases. Therefore, we hypothesize that Ptc2 inactivates a kinase to stabilize Ume6 in response to 5% CO_2_. To identify the kinase that promotes Ume6 degradation, we screened a knockout library of 80 kinases and kinase-related genes (46) for mutants that grew hyperfilamentously under 0.2% O_2_ but showed wild-type levels of filamentation under 5% CO_2_. Five mutants met the criteria in hyphal elongation (Fig. 3A). We then determined Ume6 stability in 4 putative kinase mutants under hypoxia, and the *ssn3* mutant showed stabilization of Ume6 (Fig. 3B). Ssn3 is a cyclin-dependent protein kinase, and Ssn8 is the cyclin-like component for Ssn3 (19). They are components of the mediator complex (47). In hypoxia, hyphal elongation was fully maintained (Fig. 3C), and Ume6 was stable in the *ssn3* mutant (Fig. 3D). Five percent CO_2_ had no effect on Ume6 stability and hyphal elongation in the *ssn3* mutant (Fig. 3C and D, air versus 5% CO_2_). Our data indicated that Ssn3 is the kinase for Ume6 degradation in atmospheric CO_2_.

### Phosphorylation at S437 by Ssn3 is required for CO_2_-regulated Ume6 degradation.

**FIG 4:**
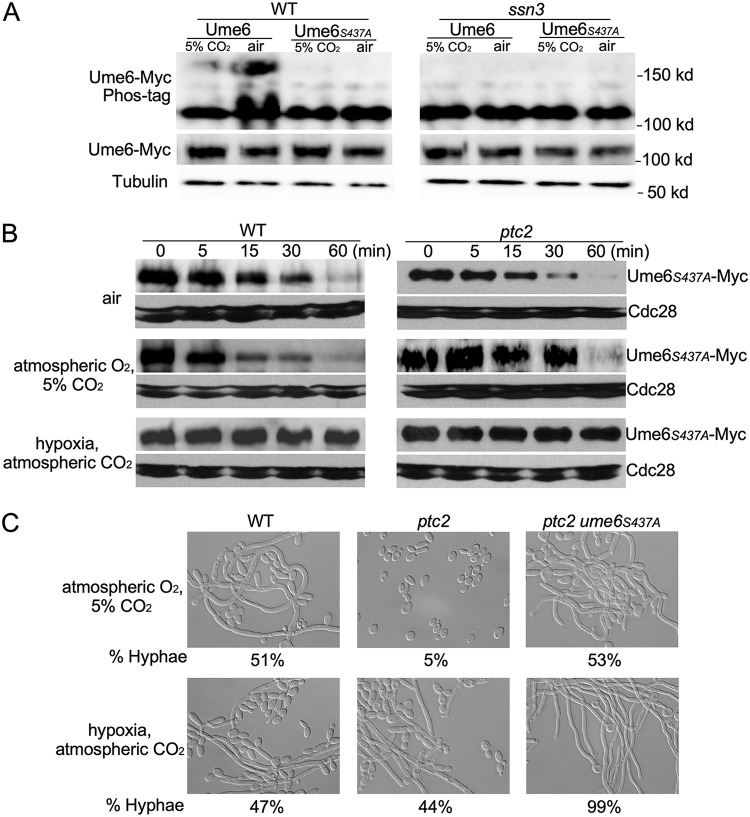
Mutating the Ssn3 phosphorylation site in Ume6 stabilizes Ume6 protein and sustains hyphal elongation under hypoxic condition. (A) Ume6 is phosphorylated at S437 in air in an Ssn3-dependent manner. Cells of wild type and *ssn3* mutant carrying Ume6-Myc or Ume6*_S437A_*-Myc were collected in air or in 5% CO_2_ at 4 h. Protein was extracted for Phos-tag gel analysis. (B) Ume6*_S437A_*-Myc stability in wild-type and *ptc2* mutant cells was monitored by *MET3* promoter shutdown under indicated conditions. (C) Morphology analysis of indicated strains was performed as described in Fig. 2A.

Given that the Ume6 degradation in response to CO_2_ requires the F-box protein Grr1, which is known to interact with phosphorylated targets (48), we predict that Ssn3 phosphorylates Ume6 in low CO_2_ to promote its degradation. Ume6 was expressed from its own promoter, and a significant portion of Ume6 showed a mobility shift in Phos-tag gels in WT cells in air, while very little Ume6 showed an upshift in 5% CO_2_ (Fig. 4A). Deletion of *SSN3* abolished the mobility shift (Fig. 4A), indicating that the phosphorylation of Ume6 in response to atmospheric levels of CO_2_ is dependent on Ssn3. Using the GPS 2.0 (Group-Based Prediction System) phosphorylation prediction system (49) with Cdk8 as the kinase and threshold set to high, 8 S/T residues in Ume6 are predicted to be phosphorylation sites of Ssn3. Among them, S437 has the highest score, 10, and S440 has the second highest score, 9.66. We mutated S437 to Ala, and examined the Ume6*_S437A_* protein in Phos-tag gels. As shown in Fig. 4A, mobility shift was not observed with the Ume6*_S437A_* under both air and 5% CO_2_, suggesting that the S437 residue is a critical phosphorylation site by Ssn3 under atmospheric CO_2_. We next expressed Ume6*_S437A_* under the *MET3* promoter to determine whether phosphorylation of S437 is essential for Ume6 degradation under atmospheric CO_2_. Using the promoter shutdown assay, Ume6*_S437A_* was partially stable in air (Fig. 4B) in comparison to Ume6 in air (Fig. 1A). Importantly, 5% CO_2_ did not increase the stability of Ume6*_S437A_*, while hypoxia was able to increase the stability of Ume6*_S437A_* (Fig. 4B). Therefore, the S437 is required for Ume6 phosphorylation and degradation under atmospheric CO_2_. We next examined Ume6*_S437A_* stability in the *ptc2* mutant, in which Ume6 is degraded similarly in atmospheric CO_2_ and 5% CO_2_ (Fig. 2B). In contrast to wild-type Ume6 in the *ptc2* mutant (Fig. 2B), Ume6*_S437A_* is similarly stable in atmospheric or 5% CO_2_ in the *ptc2* mutant, and is completely stable in hypoxia (Fig. 4B). Thus, the Ser437-to-Ala mutation made Ume6 stable in atmospheric CO_2_, and bypassed the need for Ptc2. Next, we investigated whether the S437A mutation in Ume6 could rescue the defect of the *ptc2* mutant in hyphal elongation under 5% CO_2_. We replaced both copies of *UME6* with the Ume6*_S437A_* through Crispr-Cas9 (50). As shown in Fig. 4C, hyphal development could be sustained in the *ptc2 ume6_S437A_* mutant under hypoxia conditions. Therefore, the *ume6_S437A_* is epistatic to *ptc2*. We also mutated the S440 of Ume6 to Ala in the WT and *ptc2* mutant by CRISPR-Cas9, but the *UME6_S440A_* did not affect hyphal elongation of the WT or *ptc2* mutant under both atmospheric CO_2_ and 5% CO_2_ (Y. Lu, unpublished data). Therefore, phosphorylation of Ume6 at S437 by Ssn3 is critical for Ume6 degradation in atmospheric CO_2_. Our data suggest that Ptc2 and Ssn3 play opposite roles on the phosphorylation of Ume6 to control Ume6 stability and hyphal elongation in response to CO_2_.

### Ssn3 is dephosphorylated in 5% CO_2_ in a Ptc2-dependent manner.

**FIG 5:**
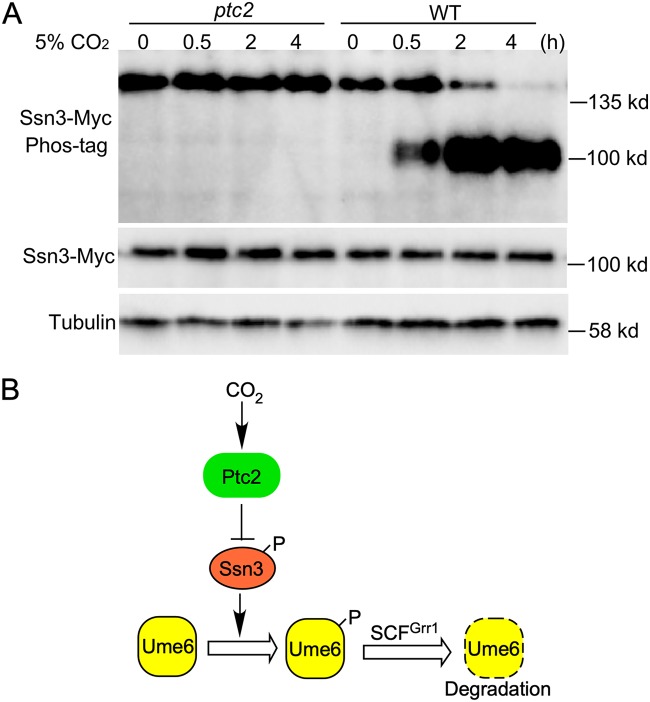
Ssn3 is dephosphorylated in 5% CO_2_ in a Ptc2-dependent manner. (A) Cells of wild type and *ptc2* mutant carrying Ssn3-Myc were collected at indicated time points after exposure to 5% CO_2_. Protein was extracted for Phos-tag gel analysis. (B) A schematic diagram depicting the CO_2_ signaling pathway mediated by the Ptc2-Ssn3 axis that controls hyphal elongation in C. albicans. A high level of CO_2_ triggers Ptc2 to dephosphorylate Ssn3. Phospho-Ssn3 promotes Ume6 phosphorylation, which leads to Ume6 degradation. The dashed circle represents degraded protein.

Since Ptc2 and Ssn3 play opposite roles on the regulation of hyphal elongation in response to CO_2_, we investigated how the CO_2_ signal is transduced to regulate Ume6 stability. In S. cerevisiae, Ptc2 is involved in the regulation of cell cycle progression as it is the main protein phosphatase acting to oppose the Cdk-activating kinase (CAK) on the activating phosphorylation site of CDK (Thr-169 of Cdc28) (24). Therefore, we hypothesize that Ptc2 inactivates Ssn3 by dephosphorylation in response to elevated CO_2_ to regulate Ume6 stability. To test this possibility, Ssn3-Myc was expressed from its own promoter in WT and *ptc2* mutant cells. Five percent CO_2_ induced Ssn3 dephosphorylation in wild-type cells, as shown by the mobility shift in Phos-tag gels (Fig. 5A). In contrast, mobility shift was not observed in the *ptc2* mutant in 5% CO_2_, indicating that Ptc2 is required for the dephosphorylation of Ssn3 in response to elevated CO_2_. Deletion of *PTC2* had no effect on the phosphorylation of Ssn3 in air, as there is no band shift exhibited at the zero point between wild-type cells and the *ptc2* mutant (Fig. 5A). Our data suggest that physiological levels of CO_2_ can induce Ptc2-mediated dephosphorylation of Ssn3. Hypophosphorylated Ssn3 fails to phosphorylate S437 in Ume6, resulting in stabilization of Ume6 to sustain hyphal development.

## DISCUSSION

Sensing of CO_2_ and rapid adaptation to changing levels of CO_2_ are an essential process in all living cells. This is particularly important for pathogenic fungi that are able to grow in a wide range of CO_2_ levels from atmospheric 0.036% to physiological 5% in the hosts. CO_2_ is hydrolyzed into bicarbonate inside the cell naturally and through the activity of carbonic anhydrase when CO_2_ concentration is low. In C. albicans, adenylyl cyclase Cyr1 acts as a bicarbonate sensor to regulate hyphal morphogenesis. Through genetic screens, we identified a new CO_2_ signaling pathway that governs Ume6 protein stability to promote hyphal elongation in C. albicans. Ptc2, a type 2C protein phosphatase, is specifically required for CO_2_-responsive hyphal elongation. High levels of CO_2_ trigger Ptc2 to dephosphorylate Ssn3. The hypophosphorylated Ssn3 fails to phosphorylate Ume6 at the S437 residue, which prevents Ume6 from being targeted by SCF^Grr1^ for ubiquitination, and therefore stabilized to promote hyphal elongation (Fig. 5B). As we previously reported (17), the stabilization of Ume6 protein is coordinately regulated by hypoxia and high CO_2_. Here we demonstrate that Ume6 stability is controlled by two E3 ubiquitin ligases in response to hypoxia and high CO_2_, respectively. A recent study by Mendelsohn et al., showed that Cdk1-Hgc1 promotes Ume6 degradation via the SCF^Cdc4^ ubiquitin ligase (51). Both SCF^Grr1^ and SCF^Cdc4^ ubiquitin ligases likely participate in Ume6 degradation, as the *Cacdc53^ts^* mutant completely blocked Ume6 degradation while *CDC4* shutdown only partially affected Ume6 degradation (51). Cdc53 is an essential protein of SCF complexes, including SCF^Grr1^ and SCF^Cdc4^. Our promoter shutdown assay for Ume6 stability was carried out at 30°C with the Ume6-myc protein that is defective in DNA binding. Therefore, the expression level of hypha-induced genes, including *HGC1,* is expected to be low under our assay condition. Cdk1/Hgc1-activated Ume6 degradation may not contribute to Ume6 stability in this study. Like Ume6, Hgc1 degradation could also be regulated by multiple signaling pathways and E3 ubiquitin ligases (Fig. S1), which explains why the *Cagrr1* deletion does not block Hgc1 degradation (42, 43). It is known that CO_2_ production is directly coupled to oxygen consumption of eukaryotic cells, and sites of hypoxia *in vivo* often contain increased levels of CO_2_. Therefore, our study may provide the underlying mechanism of how the interconnections and relationship are established between oxygen and carbon dioxide sensing with regard to fungal pathogenesis.

Our identification of the Ptc2-Ssn3 axis that governs CO_2_-responsive Ume6 stabilization and hyphal elongation provides molecular insights into fungal CO_2_ sensing. CO_2_ is a key determinant involved in fundamental biological processes, including growth, morphology, and virulence in fungi (52). Adenylyl cyclase acts as a bicarbonate sensor to promote hyphal growth in response to elevated CO_2_ levels in C. albicans (35). A recent report revealed the regulatory role of the TCA cycle in CO_2_ sensing and hyphal development through integration with the Ras1-cAMP signaling pathway in C. albicans (53). However, a pulse of activation of cAMP-PKA pathway promotes hyphal initiation, yet is not sufficient for long-lasting hyphal maintenance (14, 15). The Sch9 kinase has been shown to downregulate hyphal formation in hypoxia and high CO_2_ (54). Sch9 is also involved in the regulation of CO_2_-responsive carbonic anhydrase expression (39). However, hyperfilamentation of the *sch9* mutant was detected only during growth on agar and at low temperature. Deletion of *SCH9* resulted in even lower levels of hypha formation compared to wild-type cells in liquid media at 37°C (54), suggesting that Sch9 is unlikely to function through the same CO_2_ signaling pathway as Ssn3 in the regulation of Ume6 stability. Genome-wide analysis revealed that the transcription levels of a large number of genes are changed in C. albicans to adapt to high CO_2_. For example, genes related to the TCA cycle, genes responsive to stress and drugs, and amino acid synthesis-related genes are upregulated in 5% CO_2_. Whether these regulations occur through the Ptc2-Ssn3-mediated signaling pathway needs to be further investigated.

Ssn3, a cyclin-dependent kinase, promotes Ume6 degradation under atmospheric CO_2_ (Fig. 3). Under atmospheric CO_2_, Ume6 is phosphorylated in an Ssn3-dependent manner (Fig. 4A). Moreover, Ume6 is completely stable under atmospheric CO_2_ and hypoxia conditions in the *ssn3* mutant (Fig. 3D) or when the Ssn3-dependent phosphorylation site (S437) is mutated (Fig. 4B). Based on these results, we propose that Ssn3 is inactivated in 5% CO_2_, thus preventing Ume6 from phosphorylating at S437, which is necessary for CO_2_-responsive Ume6 degradation. C. albicans mutants defective in the Ssn3 module of mediator lead to enhanced biofilm formation (55), and a nonsynonymous mutation in *SSN3* is sufficient for regaining the ability to filament in the absence of Efg1 and Cph1 to damage macrophages (56). It is not clear if the regulatory roles of Ssn3 in filamentation in these two studies are through transcriptional regulation via the mediator complex or via regulation of Ume6 stability. In S. cerevisiae, a number of gene-specific transcriptional regulators have been defined as Ssn3 substrates, whose degradation is induced upon phosphorylation by Ssn3 (20). In response to nitrogen limitation, a decrease in Ssn3 levels leads to stabilization of two key transcription activators, Ste12 and Phd1, which promotes pseudohyphal growth in S. cerevisiae (57, 58). In this study, we did not observe a decrease in the protein level of Ssn3 when C. albicans cells were exposed to 5% CO_2_ (Fig. 5A), suggesting that inactivation of Ssn3 in 5% CO_2_ is not through downregulating *SSN3* expression. A recent study reported that cyclin C (Ssn8) is destroyed in response to oxidative stress, leading to Cdk8 (Ssn3) inactivation (59). However, *SSN8* expression is not regulated by CO_2_ levels, and overexpression of *SSN8* had no effect on CO_2_-induced hyphal elongation (Y. Lu and H. Liu, unpublished data). Therefore, Ssn3 activity is probably not regulated through changing the levels of its associated cyclin Ssn8.

Our genetic screens identified a phosphatase Ptc2 and a kinase Ssn3 as the major positive and negative regulators in CO_2_ signaling of sustained hyphal development, respectively. Ptc2 is a type 2C Ser/Thr phosphatase that is conserved in eukaryotes and involved in a large variety of functional processes. Ptc2 dephosphorylates a number of kinases, including Hog1, Ire1, and Cdc28, to repress the activity of these kinases in S. cerevisiae (24, 27, 28). It is yet to be determined if Ptc2 regulates any of these kinases in C. albicans. Here we show that, in response to 5% CO_2_, Ssn3 is dephosphorylated in a Ptc2-dependent manner. Our data suggest that Ssn3 is a downstream target of Ptc2 in this CO_2_ signaling pathway, and Ssn3 activity is inhibited upon dephosphorylation by Ptc2. This study adds an additional layer of the regulation of Ssn3 activity and provides the first example, to our knowledge, of how Ssn3 activity is regulated through changing its phosphorylation state in response to environmental cues. Given that Ptc2-mediated inhibition of Ssn3 occurs through dephosphorylation, the origin of the activating phosphorylation should be considered. Two modes of Ssn3 activation can be envisaged: autophosphorylation or phosphorylation by upstream kinases. Ssn3 may be activated by autophosphorylation, as we did not identify another kinase mutant from our screening that exhibited a similar phenotype as the *ssn3* mutant on the regulation of Ume6 stability. Such a PP2C-kinase regulatory module is also used by plants in ABA (abscisic acid) signaling, whereby ABA binding by PYR1/PYL/RCAR soluble ABA receptors inhibits PP2C phosphatases such as ABI1, ABI2, and HAB1 (60, 61), allowing serine-threonine SnRK2-type kinases (sucrose nonfermenting-1 [Snf1]-related protein kinase 2) to perform activation and phosphorylation of target proteins (62, 63). Taken together, our study elucidated a new regulatory mechanism for CO_2_ signaling in C. albicans through the Ptc2-Ssn3-medated protein phosphorylation/dephosphorylation system.

## MATERIALS AND METHODS

### Media and growth conditions.

C. albicans strains were routinely grown at 30°C in YPD (2% Bacto peptone, 2% dextrose, 1% yeast extract). Transformants were selected on synthetic medium (2% dextrose, 0.17% Difco yeast nitrogen base without ammonium sulfate, 0.5% ammonium sulfate, and auxotrophic supplements) or YPD + 200 µg/ml nourseothricin plates. Hyphal inductions were performed as follows. Strains were grown overnight in liquid YPD at 30°C, pelleted, washed twice in PBS, resuspended in an equal volume of PBS, and diluted 1:250 in YPSucrose medium (2% Bacto peptone, 2% sucrose, 1% yeast extract) with or without 10% serum at 37°C. For hyphal induction in hypoxia or 5% CO_2_, experiments were carried out using a Galaxy R170 CO_2_ incubator (Eppendorf). The oxygen and carbon dioxide concentrations were controlled by varying the concentration of nitrogen or carbon dioxide. Two hundred fifty microliters of prewarmed YPSucrose medium (buffered with citrate acid at pH 6.0) was added to each well of a 24-well plate, and 1 μl of overnight culture was inoculated into each well. The plate was placed into the incubator at 37°C immediately. After 12 h, cells were collected for morphological analysis.

### Screening for mutant defective in hyphal elongation under 5% CO_2_.

The deletion mutant library affecting 674 genes of C. albicans (34) and the wild-type reference strain SN250 were grown overnight in liquid YPD at 30°C. Thirty-five mutants grew as elongated pseudohyphae, and they were excluded from further analysis. The remaining 639 mutants and wild-type cells were diluted at 1:250 to the buffered YPSucrose (pH 6.0) medium at 37°C under hypoxia (0.2% O_2_) plus 5% CO_2_ for 12 h. Thirty-three mutants were defective in hyphal elongation under this condition; only the *ptc2* mutant was very defective in CO_2_-induced hyphal maintenance but had no defect in hyphal development in YPD + 10% serum.

### Plasmid and strain construction.

The C. albicans strains used in this study are listed in Table S1 in the supplemental material. Primer sequences are listed in Table S2. The wild-type SN250 and *ptc2* mutant were streaked on 5-fluoro-orotic acid-containing medium to generate Ura^−^ strains. Two-step PCR was used to create pMET3-UME6*_C778/785S, S437A_*-13MYC. Two pairs of primers (primers 1 and 2 and primers 3 and 4) were used to PCR amplify overlapping *UME6* fragments with the mutation in the overlapping region from the plasmid pMET3-UME6*_C778/785S_* (17). The resulting PCR products were purified and mixed as the templates for another round of PCR amplification using the primers 1 and 3, which produced the full-length *UME6_C778/785S, S437A_* sequence. The resulting mutant *UME6_C778/785S, S437A_* was inserted into the BamHI-MluI site of pPR673-MET3p (17) to generate pMET3-UME6*_C778/785S,__S437A_*-13MYC by Gibson assembly. The plasmid was digested with PmlI within the *MET3* promoter region for integration into the endogenous *MET3* locus. Both copies of *UME6* were replaced by *UME6_S437A_* using CRISPR-Cas9 (50) to construct C. albicans *UME6_S437A_* mutant strains as follows. The sgRNA (primers 5 and 6) was annealed to insert into pV1093 vector. The resulting plasmid was linearized by digestion with KpnI and SacI and was transformed into wild-type and *ptc2* mutant cells with the repair template (primers 7 and 8). The mutants were verified by sequencing.

10.1128/mBio.02320-18.2 TABLE S1 C. albicans strains used in this study. Download Table S1, PDF file, 0.3 MB. Copyright © 2019 Lu et al. 2019 Lu et al. This content is distributed under the terms of the Creative Commons Attribution 4.0 International license.

10.1128/mBio.02320-18.3 TABLE S2 Primers used in this study. Download Table S2, PDF file, 0.1 MB. Copyright © 2019 Lu et al. 2019 Lu et al. This content is distributed under the terms of the Creative Commons Attribution 4.0 International license.

A 1.2-kb PCR product (primers 9 and 10) containing the C-terminal *SSN3* coding region was inserted into the BamHI-MluI site of pPR673. The resulting plasmid was digested with SacI to target integration into its own locus to express Ssn3-13Myc.

### Promoter shutdown assays.

C. albicans strains containing Ume6*_C778/785S_*-Myc (shown as Ume6-Myc) or Ume6*_C778/785S,S437A_*-Myc (shown as Ume6*_S437A_*-Myc) under the regulation of the *MET3* promoter were grown in SCD (−Met, −Cys) for 2 h to induce their expression at room temperature. Twenty-five milliliters of medium was transferred from the culture to a petri dish (150 × 15 mm) and placed into air, a hypoxic chamber, or a CO_2_ incubator as indicated. After incubation at 30°C for 4 h, 5 mM methionine was added to shut off the promoter. Aliquots were collected after the times indicated, and protein levels were analyzed via Western blotting.

### Phos-tag SDS-PAGE.

Phosphorylation states of Ume6-Myc and Ssn3-Myc were examined using Phos-tag SDS-PAGE, which is a phospho-affinity SDS-PAGE developed by Kinoshita et al. (64). Phos-tag acrylamide was purchased from Wako Chemicals (Osaka, Japan). Separating gels were made by copolymerization of acrylamide with Phos-tag acrylamide. Phos-tag SDS-PAGE was performed on 6% polyacrylamide gels containing 50 μM Phos-tag acrylamide and 100 μM MnCl_2_ in 10-mA current at 4°C. The separated proteins were transferred to PVDF membranes. Immunoreaction of the membrane was then carried out.
