# Supplementary figures and images for: CO2 Signaling through the Ptc2-Ssn3 Axis Governs Sustained Hyphal Development of Candida albicans by Reducing Ume6 Phosphorylation and Degradation
Source: mBio. 2019 Jan 15;10(1):e02320-18. doi: 10.1128/mBio.02320-18 (PMC6336421; doi:10.1128/mBio.02320-18)

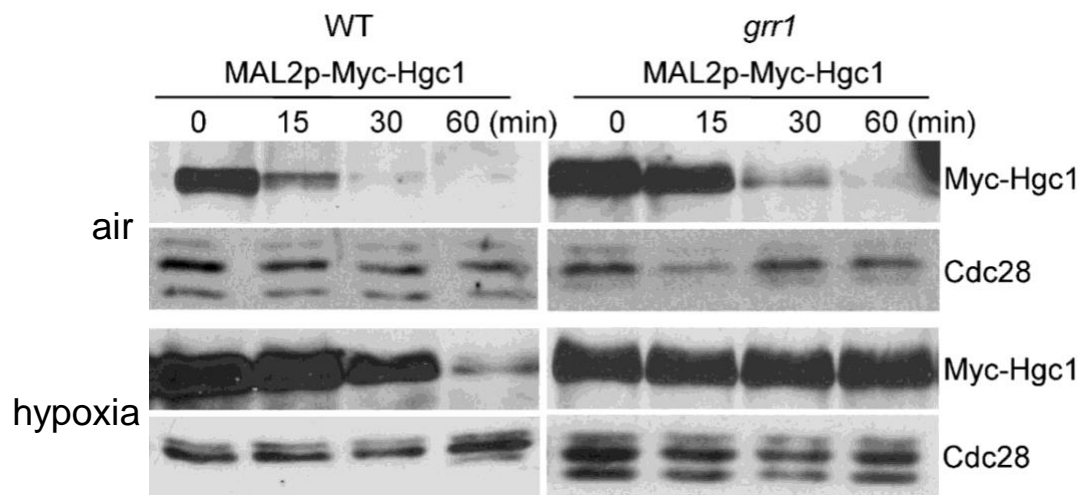

Fig S1. Hgc1 is stabilized in hypoxia in the *grr1* mutant.

Supplement: FIG S1 [file mBio.02320-18-sf001.pdf]
