# Supplementary material for: CO2 Signaling through the Ptc2-Ssn3 Axis Governs Sustained Hyphal Development of Candida albicans by Reducing Ume6 Phosphorylation and Degradation
Source: mBio. 2019 Jan 15;10(1):e02320-18. doi: 10.1128/mBio.02320-18 (PMC6336421; doi:10.1128/mBio.02320-18)
Supplement: TABLE S1 [file mBio.02320-18-st001.pdf]

Table S1. *C. albicans* stains used in this study

Strain	Parent / background	Genotype	Source
SC5314		Wild type	(1)
CAI4	SC5314	*ura3::1 imm434/ura3::1 imm434*	(1)
BWP17	SC5314	*ura3*::*1 imm434/ura3*::*1 imm434 his1*::*hisG/his1*::*hisG arg4*::*hisG/arg4*::*hisG*	(2)
HLY4176	CAI4	*ura3*::*1 imm434/ura3*::*1 imm434 MET3/met3*::*MET3p-UME6*$_{C778/785S}$*-13MYC-URA3*	(3)
HLY3578	BWP17	*ura3*::*1 imm434/ura3*::*1 imm434 his1*::*hisG/his1*::*hisG arg4*::*hisG/arg4*::*hisG ADE2/ade2*::*MAL2p-MYC-HGC1-URA3*	(4)
YLC12	HLY4176	*ura3*::*1 imm434/ura3*::*1 imm434 MET3/met3*::*MET3p-UME6*$_{C778/785S,\ S437A}$*-13MYC-URA3*	This study
YLC13	SN250	*UME6*$_{S437A}$*::UME6*$_{S437A}$ *ENO1*::CAS9-*SAT1*	This study
SN250	SC5314	*his1Δ/his1Δ, leu2Δ::C.d. HIS1/leu2Δ::C.m. LEU2, arg4Δ/arg4Δ, URA3/ura3Δ::imm434 IRO1/iro1Δ::imm434*	(5)
ptc2	SN250	*his1Δ/his1Δ, leu2Δ::C.d. HIS1/leu2Δ::C.m. LEU2, arg4Δ/arg4Δ, URA3/ura3Δ::imm434 IRO1/iro1Δ::imm434 ptc2Δ::C.d. HIS1/ptc2Δ::C.m. LEU2*	(5)
YLC15	SN250	*his1Δ/his1Δ, leu2Δ::C.d. HIS1/leu2Δ::C.m. LEU2, arg4Δ/arg4Δ, ura3Δ /ura3Δ::imm434 IRO1/iro1Δ::imm434 SSN3:: SSN3-13MYC -URA3*	This study
HLY4080	BWP17	*ubr1*::*ARG4/ubr1*::*HIS1 ura3*::*1 imm434/ura3*::*1 imm434 his1*::*hisG/his1*::*hisG arg4*::*hisG/arg4*::*hisG*	This study
HLY3586	SN148	*ura3*::*imm434/ura3*::*imm434 iro1*::*imm434/iro1*::*imm434 grr1*::*C.d.HIS1/grr1*::*C.m.LEU2 arg4Δ/arg4Δ leu2Δ/leu2Δ his1Δ/his1Δ*	(4)

References

1. Fonzi, W. A. & Irwin, M. Y. (1993) *Genetics* **134,** 717-28.
2. Wilson, R. B., Davis, D. & Mitchell, A. P. (1999) *J Bacteriol* **181,** 1868-74.
3. Lu, Y., Su, C., Solis, N. V., Filler, S. G. & Liu, H. (2003) *Cell Host & Microbe* **14**, 499-509
4. Wang, A., Lane, S., Tian, Z., Sharon, A., Hazan, I. & Liu, H. (2007) *Eukaryot Cell* **6,** 253-61.
5. Noble, S, M., French, S., Kohn, L, A., Chen, V., & Johnson, A, D. (2010) *Nat Genet* **42,** 590-8
