# Supplementary material for: CO2 Signaling through the Ptc2-Ssn3 Axis Governs Sustained Hyphal Development of Candida albicans by Reducing Ume6 Phosphorylation and Degradation
Source: mBio. 2019 Jan 15;10(1):e02320-18. doi: 10.1128/mBio.02320-18 (PMC6336421; doi:10.1128/mBio.02320-18)
Supplement: TABLE S2 [file mBio.02320-18-st002.pdf]

Table S2. Primers used in this study

Primer	Sequence
1	AAATACCCTCCCCAGAAAACGGATCCATGATTACCCATATGGTTAC
2	GTACGTGTGATGATGATGATGCTGATGGTTGATATATATGATGTGG
3	GTTCACCGTTAATTAACCCGGGAACGCGTCGATCATTGGTTATATCATTAC
4	CAACCATCAGCATCATCATCATCACACGTACGTCACAATAAAATCAC
5	ATTTGCTACCAACTATATATACTACG
6	AAAACGTAGTATATATAGTTGGTAGC
7	ATGCTTAATTCCACTTTACCATTATCCAAGTCTACTGCACATCATATATATCAACCAT
8	GTGATTTTATTGTGACGTACGTGTGATGCTGATGATGCTGATGGTTGATATATATG ATG
9	GGGAACAAAAGCTGGGTACCGGGCCCGCATTGTTCTGCTCATAGAGAC
10	CTAGAAAGTATAGGAACTTCCTCGAGGTAACCTCAAAGAGAACCATCC
11	GGAAGATCTCGACGCGTACAGATATCGACTACAAAGACCATGACGG
12	CGGGGTACCCTACTTGTCATCGTCATCCT
13	CAACAAATACAAAAACAAGGAGATCTATGGGTCAAATCCTTTCACAAC
14	GGTCTTTGTAGTCGATATCTGTACGCGTAACTTCATCATCTATTTTTTCAG
